# A Low Glycaemic Index Diet Incorporating Isomaltulose Is Associated with Lower Glycaemic Response and Variability, and Promotes Fat Oxidation in Asians

**DOI:** 10.3390/nu9050473

**Published:** 2017-05-09

**Authors:** Christiani Jeyakumar Henry, Bhupinder Kaur, Rina Yu Chin Quek, Stefan Gerardus Camps

**Affiliations:** 1 Clinical Nutrition Research Centre (CNRC), Singapore Institute for Clinical Sciences (SICS), Agency for Science, Technology and Research (A*STAR) and National University Health System, Centre for Translational Medicine, 14 Medical Drive #07-02, MD 6 Building, Yong Loo Lin School of Medicine, Singapore 117599, Singapore; Bhupinder_Kaur@sics.a-star.edu.sg (B.K.); Rina.Quek-Yu-Chin1@student.lshtm.ac.uk (R.Y.C.Q.); Stefan_Camps@sics.a-star.edu.sg (S.G.C.); 2 Department of Biochemistry, National University of Singapore, 8 Medical Drive, Singapore 117596, Singapore

**Keywords:** sucrose, isomaltulose, glycaemic index, Asians, whole body calorimeter, indirect calorimetry, continuous glucose monitoring, glycaemic response, substrate oxidation

## Abstract

Low glycaemic index (GI) foods minimize large blood glucose fluctuations and have been advocated to enhance fat oxidation and may contribute to weight management. We determined whether the inclusion of isomaltulose compared to sucrose in a low/high GI meal sequence can modulate the glycaemic response and substrate oxidation in an Asian population. Twenty Chinese men (body mass index (BMI): 17–28 kg/m^2^) followed a 24 h low GI (isomaltulose, Palatinose^TM^) or high GI (sucrose) diet in a randomized double-blind, controlled cross-over design. Treatment meals included dinner (day 1), breakfast, lunch, and snack (day 2). Continuous glucose monitoring provided incremental area under the curve (iAUC) and mean amplitude of glycaemic excursion (MAGE) and 10 h indirect calorimetry (whole body calorimeter) (day 2) provided energy expenditure and substrate oxidation. Our results demonstrated that the low GI diet resulted in lower 24 h glucose iAUC (502.5 ± 231.4 vs. 872.6 ± 493.1 mmol/L; *p* = 0.002) and lower 24 h glycaemic variability (MAGE: 1.67 ± 0.53 vs. 2.68 ± 1.13 mmol/L; *p* < 0.001). Simultaneously, 10 h respiratory quotient increased more during high GI (*p* = 0.014) and fat oxidation was higher after low GI breakfast (*p* = 0.026), lunch (*p* < 0.001) and snack (*p* = 0.013). This indicates that lower GI mixed meals incorporating isomaltulose are able to acutely reduce the glycaemic response and variability and promote fat oxidation.

## 1. Introduction

Asia has the unenviable reputation as being the epicenter for type 2 diabetes as the Asian phenotype has been shown to be more susceptible to diabetes than Caucasians [1,2]. More significantly, the transition from prediabetes to diabetes is more dramatic and severe in Asians [3]. There is good evidence to suggest that the consumption of low glycaemic index (GI) foods reduces glycaemic response and minimizes large post-prandial fluctuations in blood glucose levels which can trigger more oxidative stress and are also considered a risk factor in the onset for type 2 diabetes and impaired glucose tolerance [4,5,6].

There is increasing interest in the use of low GI foods in the management of type 2 diabetes and obesity [7]. Overweight and obesity are driving the global diabetes epidemic, and a rapid increase in the prevalence of overweight and obesity was also reported in many Asian countries, including Singapore [8]. A low GI diet has been advocated by many to not only reduce glycaemic response and variability but also influence appetite control and enhance fat oxidation [6,7,9]. Compared to low GI foods, high GI foods result in a higher glucose response and correspondingly induce a higher insulin response which, in turn, inhibits lipolysis and switches energy use to carbohydrates instead of fatty acids [7,10,11]. It is hypothesized that this favours extra fat storage and impairs weight control. Conversely, a lower insulin response after low GI foods has been hypothesized to increase fat oxidation and prolong satiety and fullness [7,12]. A higher fasting respiratory quotient (RQ) corresponds to lower fat oxidation and higher carbohydrate oxidation and has been associated with higher prospective weight gain and fat storage [13,14]. During exercise following low GI meals, increased fat oxidation and reduced postprandial blood glucose was shown in men [15] and women [16]. Moreover, a significant shift in substrate utilization from CHO to fat was observed when a low GI meal, compared to a high GI meal, was consumed before exercise [17,18]. These studies have assessed the effect of GI of a meal consumed before or after exercise on metabolic and biochemical parameters.

Numerous ingredients have been reported to reduce glycaemic response of foods; these include beta-glucan, polyols, fiber, polyphenols, and isomaltulose [19,20,21,22,23]. Isomaltulose (PalatinoseTM) is a disaccharide composed of glucose and fructose linked by an α-1,6-glycosidic bond, in contrast to sucrose where the linkage is α-1,2-glycosidic bond. The low GI of isomaltulose results from the slower hydrolysis of the α-1,6-glycosidic bond by the sucrase–isomaltase complex which is located in the wall of the small intestinal cells. As a result, the rate of absorption of isomaltulose is relatively slow, but still results in full absorption of glucose and fructose [24,25]. Previous studies have shown that isomaltulose can improve glycaemic responses in both diabetic and non-diabetic individuals [25,26,27,28]. Van Can et al. showed an acute attenuation of the glycaemic and insulinaemic response in overweight subjects with impaired glucose tolerance, while also showing less decline in fat oxidation after isomaltulose consumption [27]. In Japanese subjects, Okuno et al. showed an improvement in insulin sensitivity after 12 weeks of isomaltulose supplementation compared to sucrose [28].

In this study, we simultaneously measured the dynamic response in both glycaemic response and substrate oxidation up to 42 h during an acute randomized trial to investigate the metabolic effects of the inclusion of isomaltulose or sucrose in a daily meal sequence in a free living Asian population. The study population was homogeneous with regards to ethnicity (Chinese) and gender (male), though representing a wide range of BMI. In the present study, the glycaemic load of the entire meal was modulated by adding sucrose or isomaltulose to create a high or low GI diet. We hypothesize that the inclusion of isomaltulose to develop low GI meals will lower the glycaemic response and variability and will result in higher fat oxidation.

## 2. Materials and Methods 

### 2.1. Subjects

Twenty healthy Chinese male subjects were recruited by a variety of methods, which included flyers, online advertisement, and personal communication around the University campus. Subjects underwent an initial screening and measurements, included anthropometry (height, weight, waist, and hip circumference), fat percentage via air displacement plethysmography (BodPod, Life Measurements Inc., Concord, CA, USA), blood pressure, resting heart rate, fasting blood glucose, and %HbA1C. Additionally, questionnaires on general health, physical activity [29] and eating behaviour [30] would have to be completed. Twenty men were assessed for eligibility and all fulfilled the following criteria: Chinese, male, between 21–40 years, not glucose-6-phospahate dehydrogenase deficient, not allergic to the test foods, not participating in sports at a competitive and/or endurance level, body mass index (BMI) between 17 and 28 kg/m^2^, normal blood pressure (<140/80 mmHg), not intentionally restricting food intake, and fasting blood glucose lower than 6 mmol/L. All subjects followed both the treatment diets and none dropped out or were excluded from the analysis.

The study was conducted at the Clinical Nutrition Research Centre (CNRC), Singapore. Written informed consent was obtained from all eligible participants before commencement and the research procedures and trial protocols were followed in accordance to the good clinical practice guidelines and with the ethical standards in concordance to the Declaration of Helsinki, 1983. Ethical approval of all procedures involving human subjects was obtained from the National Healthcare Group Domain Specific Review Board (NHG DSRB). This trial was registered as NCT 03031886 (Clinicaltrials.gov).

### 2.2. Study Protocol

**Figure 1:**
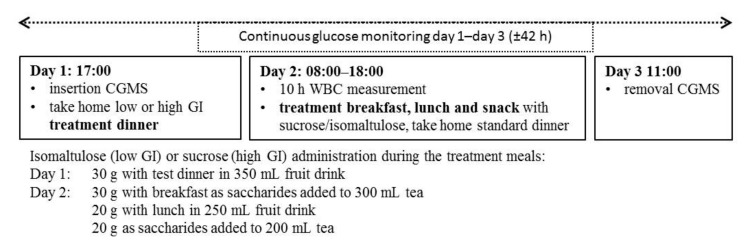
Schematic overview of the study design.

The study consisted of two dietary treatments in a randomized double-blinded, controlled cross-over design: a low glycaemic index (LGI) and high glycaemic index (HGI) diet. Subjects attended both sessions where they followed a 24 h LGI or HGI diet depending on the randomization. Manipulated treatment meals included: dinner on day 1 and breakfast, lunch, and an afternoon snack on day 2; dinner on day 2 would be standardized over the two dietary treatments. The two test sessions of three consecutive days each were separated by a wash-out period of at least five days. Each test session would start at 17:00 on day 1 and end on day 3 at 11:00 consisting of a 42 h continuous glucose measurement (CGM) spanning the three days and a 10 h measurement of energy expenditure and substrate oxidation in a whole body calorimetry (WBC) room on day 2. On days 1 and 2, subjects were allowed to leave the research centre at 18:00 and on Day 3 at 11:00. Figure 1 shows a schematic overview of the study design. Subjects had to log their total food intake on day 1 and record their physical activity for all 3 days. In addition, subjects were instructed to refrain from strenuous physical activity and were only to eat the provided foods throughout test sessions. Online computer software was used for simple randomization of the sequence of the treatment diets [31].

### 2.3. Treatment Meals

The study consisted of a low GI and high GI treatment diet and treatment meals included: dinner on day 1 and breakfast, lunch, and an afternoon snack on day 2 (Table 1). The glycaemic load of the treatment meals was modulated by adding sucrose (HGI) or isomaltulose (PalatinoseTM) (LGI). The daily meals provided to the subjects matched their daily energy requirements based on the measured basal metabolic rate of the subject multiplied by a low physical activity level of 1.5 (27). The standardized dinner consisted of a ready-to-eat teriyaki chicken with rice, a soda drink and one mango-flavoured jelly pudding and reflected a typical local rice-based meal accompanied with a drink and dessert (energy% carbohydrate: 64%, energy% protein: 20%, energy% fat: 16%).

### 2.4. Glycaemic Measurements

Continuous glucose monitoring (CGM) (iPro™2 Professional CGM-Medtronic MiniMed, Northbridge, CA, USA) was used to measure glycaemic response, defined as the primary outcome. The insertion of the sensor was performed on day 1 at 17:00 and the sensor was removed on day 3 at 10:00. Data was collated and processed using online software (Medtronic Diabetes CareLink iPro) [32]. The data reported in this paper represent interstitial glucose readings recorded every 5 min for up to 42 h. During each test session, the CGM sensor was calibrated against finger-stick blood glucose measurements four times a day before every meal and before sleeping using a blood glucose meter (OneTouch^®^Ultra^®^2, LifeScan, Inc., Milpitas, CA, USA). A cross-over design with a minimum of eight subjects would be sufficient to detect a 15% change in area under the glucose curve (24 h) with a power of 0.85 at a significance level of 0.05 [33].

### 2.5. Energy Expenditure and Substrate Oxidation

Energy expenditure, respiratory quotient (RQ) and substrate oxidation were measured using a dual room WBC facility based on the system described by Schoffelen et al. [34,35,36]. Each WBC room is an open circuit, airtight indirect calorimeter with a total volume of 13.5 m^3^, furnished with a single-bed, a foldable chair, a bureau with a built-in sink, deep-freeze toilet (Special Product, Mulders), a colour television, an alarm clock, a radio, a telephone, a laptop, WIFI connection, and an automated intercom for communication between the researcher and the participant. It is built to mimic a normal room with two windows for visual contact between the researcher and participant.

During the test session, gaseous exchanges were measured continuously as oxygen consumption and carbon dioxide production through differences between the inlet and outlet oxygen (O_2_) and carbon dioxide (CO_2_) concentrations. Oxygen concentration was measured using a paramagnetic O_2_ analyser (Model AO2020, module Magnos206, ABB Automation GmbH, Frankfurt am Main, Germany), while carbon dioxide concentration was measured using an infrared photometer (Model AO2020, module Uras26, ABB Automation GmbH, Frankfurt am Main, Germany). The air samples are measured in an automated sequence and alternated with a calibration span gas (18% O_2_, 0.8% CO_2_, and balance nitrogen) and zero (100% nitrogen) gases [34]. Gaseous exchanges were measured under standard temperature, pressure, and dry (STPD). The accuracy of the WBC chambers was routinely tested through the combustion of a known amount of methanol. The accuracy of O_2_ and CO_2_ measurements in our WBC facility were: O_2_ = 100.6% ± 0.5% (chamber 1) and 100.9% ± 0.4% (chamber 2), and CO_2_ = 99.2% ± 0.5% (chamber 1) and 99.7% ± 0.5% (chamber 2), while the coefficient of variation was 3.0% (*n* = 21) for repeated 30-minute RMR measurements with our WBC facility.

Energy expenditure was calculated based on volume of O_2_ consumption (VO_2_) and CO_2_ production (VCO_2_) using the Weir equation [37]. Substrate oxidation and respiratory quotient were calculated from urinary nitrogen excretion, oxygen consumption and carbon dioxide production [38]. Urine samples were collected in the WBC over 10 h in a 3 L 24-h urine collection container (Urisafe^®^, Simport Scientific, Beloeil, QC, Canada). The total volume of urine over 10 h was measured and a representative urine sample was stored for nitrogen analysis. Nitrogen content (%) was measured using the copper catalyst Kjeldahl method (AOAC Official Method 984.13). Protein oxidation (g/min) was calculated by multiplying 10 h urinary nitrogen (g) by 6.25 and converted to per minute values. CHO oxidation and fat oxidation were calculated by using the following equations based on the volumes of O_2_ consumed and CO_2_ produced in oxidation of glucose, fat, and protein as published by Frayn [38]:

CHO oxidation (g/min) = −3.21 × O_2_ (L/min) + 4.55 × CO_2_ (L/min) − 2.87 × N (g/min) fat oxidation (g/min) = 1.67 × O_2_ (L/min) − 1.67 × CO_2_ (L/min) − 1.92 × N (g/min)

### 2.6. Statistics

The participants and researchers involved in the measurements and analysis and of the data were blinded to the randomized treatment sequence until the end of the study period. All statistical analyses were performed using SPSS version 23 (IBM Corp, Armonk, NY, USA). Data and figures were processed in a Microsoft Excel (Microsoft, Redmond, WA, USA). Values were presented as means ± standard deviation (SD), unless otherwise stated. Prior to statistical analysis, the normality of the data was assured using the Shapiro-Wilks test.

The primary outcome of this study was to determine how the inclusion of isomaltulose (LGI) compared to sucrose (HGI) in a diet sequence (dinner, breakfast, lunch, and snack) impacts 24 h glycaemic response and variability and energy regulation. First, the baseline glucose value for each subject was determined from the two-hour average CGM interstitial glucose readings before basal metabolic rate (BMR) measurement in a fasted state on day 2 and used to calculate the change in glucose level for all 42 h. Glycaemic response was expressed as the incremental area under the curve (iAUC) and calculated using the trapezoidal rule and the change in glucose above baseline [39,40]. Total glucose AUC was calculated using the absolute glucose values. Second, mean amplitude of glycaemic excursion (MAGE) was assessed as an indicator for glycaemic variability during the day [41,42,43]. MAGE was calculated using EasyGV software [44], with this software being extensively reviewed [45]. Third, baseline substrate oxidation and RQ were calculated from 45 min steady-state measurement of BMR in a fasted state and used to assess 10 h changes from baseline. Subsequently, substrate oxidation and RQ values were calculated for 15 min before each meal and used to calculate meal specific post-prandial changes. A paired *t*-test was performed to test the differences in the glycaemic response (one-tailed), MAGE, and substrate oxidation (two-tailed) between LGI and HGI treatment diets, and alpha (α) was set at 0.05 for statistical analyses.

## 3. Results

Subject characteristics at baseline are presented in Table 2.

**Figure 2:**
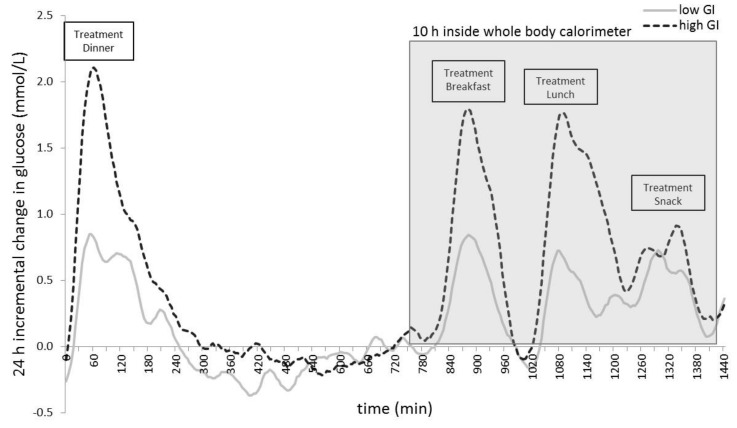
Mean change in glucose concentrations from baseline over 24 h (*n* = 20) for the two dietary treatments (dotted line = high glycaemic index (GI), full line = low GI). Twenty-four hours from before the treatment dinner until before the standardized dinner. Manipulated treatment meals are indicated.

Low and high GI treatment resulted in a significantly different response in most glycaemic parameters as presented in Figure 2 and Table 3. Twenty-four hour iAUC in Table 3 corresponds with the visual representation in Figure 1 and shows a higher glucose response during the 24 h on a high GI diet. The low GI diet treatment resulted in a lower 24 h glucose iAUC (502.5 ± 231.4 vs. 872.6 ± 493.1 mmol/L; *p* = 0.002) with a lower postprandial glucose iAUC after breakfast and lunch (*p* < 0.001). Moreover, the low GI diet treatment resulted in lower 24 h glycaemic variability (MAGE: 1.67 ± 0.53 vs. 2.68 ± 1.13 mmol/L; *p* < 0.001).

The high GI diet treatment resulted in a significantly higher 10 h increase in RQ (*p* = 0.014), a significantly higher 10 h increase in carbohydrate oxidation (*p* < 0.001) and a trend for a larger 10 h decrease in fat oxidation compared to low GI (*p* = 0.052) (Table 4). Meal-specifically, the low GI diet treatment resulted in a significantly smaller increase in carbohydrate oxidation while favouring a smaller decrease in fat oxidation after breakfast (*p* = 0.026) and lunch (*p* < 0.001) compared to the high GI diet treatment (Table 5).

**Figure 3:**
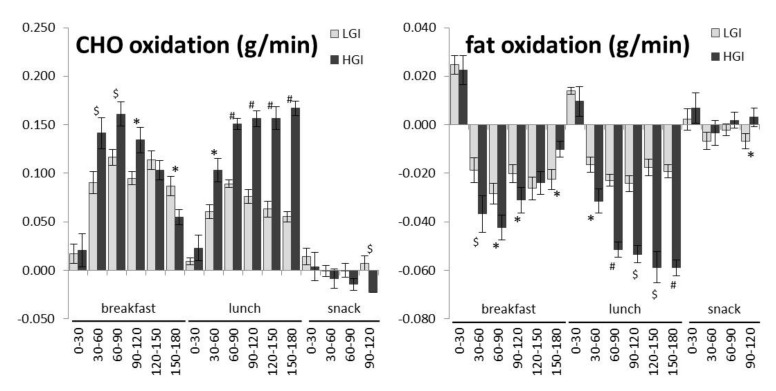
Changes in carbohydrate (CHO) oxidation and fat oxidation compared to meal-specific baselines (*n* = 20). Values are the averages per 30 min and error bars represent the SEM. * *p* < 0.05, ^$^ *p* < 0.01, ^#^ *p* < 0.001 (paired Student’s *t*-test). LGI: low glycaemic index; HGI: high glycaemic index.

The change in carbohydrate and fat oxidation compared to the meal-specific baseline is presented in Figure 3. 30 min averages show a smaller decrease in fat oxidation 30–120 min after breakfast and 30–180 min after lunch for the low GI diet.

## 4. Discussion

The current study investigated the effects of a 24 h low vs. high glycaemic index diet on the glycaemic response and substrate metabolism in normal and overweight Chinese subjects. The novelty of this study was the simultaneous and continuous measurement of these two metabolic parameters in healthy Chinese subjects to investigate the acute effect of including isomaltulose to mixed meals compared to sucrose. The addition of isomaltulose at every meal event is a unique way in which the glycaemic response of the foods may be significantly diminished. A low GI diet proved to be able to lower the blood glucose response and glycaemic variability while at the same time favouring higher fat oxidation. The effects were most pronounced after breakfast and lunch. The analogous response reflects a high likelihood that insulin is modulated by a lower GI diet and stimulates higher fat oxidation.

Our study showed that the addition of isomaltulose or sucrose to a low or high GI meal was able to acutely lower 24 h glycaemic response and 24 h glycaemic variability. This attenuation of glucose response was also observed during the 10 h period in the whole body calorimeter. These results are in line with shorter acute trials showing an attenuated glucose and insulin response after isomaltulose co-ingestion compared to sucrose in Caucasian subjects [25,27]. Additionally, continuous glucose monitoring allowed us to look in detail to glycaemic excursions throughout the day in an Asian population over a prolonged 42 h period. To our knowledge a limited number of studies have measured 24 h continuous blood glucose profiles in healthy, non-diabetic subjects with even fewer studies performed on normal weight and overweight Asians. After consumption of a low GI dinner, participants showed a lower glycaemic response throughout the night and the attenuated glucose response was prolonged after consumption of low GI breakfast, lunch and a snack on the following day. Although a standard dinner was consumed, the glycaemic response post-prandially and nocturnally was lower when a low GI diet was pre-consumed. The carry-over effect of the low GI diet is in line with the “second-meal effect” previously found; some studies have shown that low GI foods ingested at breakfast improved glycaemia following a subsequent standardized lunch meal [46,47,48]. Consuming foods that elicit a second meal effect may help towards the maintenance of low blood glucose concentrations in the short and medium term and thereby reduce demands on the insulin mediated blood glucose regulatory systems. In this study, a carry-over effect of a 24 h low GI diet was observed during a subsequent dinner and night. Normalizing nocturnal glycaemia will help to reduce fasting glucose concentrations and potentially reduce cardiovascular disease (CVD) risk in healthy people [49]. Consumption of the low GI diet also reduced 24 h glycaemic variability which is considered a risk factor in the onset of type 2 diabetes [4]. It is evident that also in young, healthy Chinese males within a large range of BMI, low GI meals are beneficial for glycaemic health. The results support and build on the evidence that suggest that the consumption of low GI foods is beneficial for glycaemic control; this might be especially significant in diabetic people [5,6,33].

For the first time in an Asian population, substrate oxidation during a low and high GI diet consumption was measured using and dual room whole body calorimeter unit simultaneous to the continuous glucose monitoring. As expected, there is a meal induced increase in carbohydrate oxidation and decrease in fat oxidation compared to baseline and as hypothesized, the 10 h respiratory quotient increased more during high GI, indicating relatively lower fat oxidation and higher carbohydrate oxidation. During the 10 h whole body calorimeter measurement, a higher increase in carbohydrate oxidation and a trend for a larger decrease in fat oxidation were seen during the sucrose containing high GI diet, which is line with current literature [50]. Meal specific analysis showed a significant difference in substrate oxidation in favour of fat oxidation after the low GI, isomaltulose enriched meals compared to sucrose. Similarly to the glycaemic response this was more profound after breakfast and lunch but evident throughout the stay in the whole body calorimeter. These results are in line with van Can et al. who showed an attenuated postprandial inhibition of fat oxidation after lunch after isomaltulose co-ingestion during breakfast and lunch [27]. Additional analysis of carbohydrate and fat oxidation per half hour shows that substrate oxidation is highly variable over time, more so during the high GI diet which is in line with larger fluctuations in blood glucose. From 30 to 120 min after breakfast, there is a higher carbohydrate oxidation during high GI compared to low GI, however from 120 min after breakfast a lower carbohydrate oxidation can be observed which can be expected for a high GI diet [51]. Meal-specific baselines were calculated to account for the differences of substrate oxidation between the conditions at the start of each meal. From the data it is clear that the lunch resulted in a larger decrease in fat oxidation during the high diet from 30 min all the way until the afternoon snack. The snack was relatively small compared to lunch and resulted only in a small effect. Over the full 10 h, and after each meal, the isomaltulose-modulated meals were able to shift substrate oxidation in favor of fat oxidation; this is in line with existing data [7].

Stevenson and colleagues have shown that meals composed of low GI carbohydrates were able to reduce postprandial plasma glucose and increase fat oxidation during subsequent exercise [15,16]. Studies also showed that isomaltulose supplementation continuously over several months can improve glycaemic responses in both diabetic and non-diabetic, Caucasians and Japanese Asians [26,28]. Furthermore, van Can et al. reported, less decline in fat oxidation during isomaltulose co-ingestion in overweight, impaired glucose tolerant subjects and improved glycaemia and insulin sensitivity [27]. Now, we have shown that even in a sedentary state, the consumption of a (isomaltulose including) low GI diet acutely resulted in better glucose control while simultaneously promoting fat oxidation in healthy Chinese. This is in agreement with previous studies that showed lower blood glucose and increased fat oxidation when LGI or HGI carbohydrates were used [52,53]. Several authors have reported better weight control or reduced fat deposition when fed a LGI diet [7,54,55] but the exact mechanism is not known. Now, the concomitant measurement of blood glucose and substrate oxidation enables us to quantitate both glucose flux and fat tissue accretion and demonstrates that increased fat oxidation is the driver of reduced fat deposition when fed LGI. Along the same explanation, it has been shown previously, that higher increase in insulin and inhibition of glucagon shortly after a HGI meal results in a notably higher insulin:glucagon ratio compared to LGI. This promotes the uptake of carbohydrates and fat by liver and muscle after HGI meals. This is supported by our results indicating lower fat oxidation after HGI meals. It also provides a mechanistic explanation why a low GI meal with the inclusion of isomaltulose facilitates fat oxidation and supports the systematic observation that consuming a low GI diet facilitates weight management.

Some researchers found that the GI of mixed meals was unable to modify fuel partitioning in sedentary obese women [56,57]. Diaz and colleagues found that carbohydrate and fat oxidation was not modified by glycaemic meal characteristics in the five hours following breakfast and lunch and concluded that the lack of effect of serum insulin response on fat oxidation may be due to the short-time period in which serum insulin concentration was maintained at a quantitatively higher level when comparing HGI to LGI meals [56]. The main differences between these studies and our study were the use of obese subjects who might be less susceptible to fat oxidation, and these subjects were overfed a high-carbohydrate load irrespective of whether the meals were LGI or HGI. Recently, Bosy-Westphal et al. hypothesized that a diet-induced hyperinsulinemia may lead to higher fat storage only during a positive energy balance. Additionally, people may be at risk towards more fat storage when physically inactive due to increased insulin sensitivity in adipose tissue [58]. Hence, in the case of a sedentary lifestyle, a low GI diet might be even more beneficial to curb weight gain.

A minor limitation was that the protein oxidation could not be measured over a continuous 24 h period and did not provide detailed hourly changes. It was, therefore, necessary to estimate the average protein oxidation based on the 10 h cycle in the WBC. The rate of protein oxidation was assumed to be constant during the time in the WBC [59]. Although the study was performed in male subjects alone, previous studies have demonstrated a similar effect in mixed groups including men and women [26,28]. Serum insulin measurements could have significantly strengthened the study and further support the link between glycaemic response and fuel utilization. Despite these limitations, our study demonstrates how the inclusion of isomaltulose, as compared to sucrose, with mixed meals can acutely increase fat oxidation mediated via a lowering of blood glucose. For future research, the changes in glycaemic response and substrate oxidation after the prolonged consumption of low GI diets developed with isomaltulose present an interesting opportunity.

## 5. Conclusions

Unique to our study was the simultaneous measurement of blood glucose and substrate oxidation using a whole-body calorimeter when subjects were fed low or high GI meals modulated as such by including isomaltulose or sucrose. This study demonstrates that including isomaltulose to develop low-GI mixed meals is able to acutely moderate glycaemic response while promoting fat oxidation over carbohydrate oxidation during relative inactivity in Chinese subjects when compared to high GI meals. Whilst the link between blood glucose levels and fat oxidation has been demonstrated in this study, further research is necessary to quantitate how insulin and other hormones may influence tissue oxidation in humans. The consistent observation that Asians living on a high glycaemic, high carbohydrate diets are susceptible to weight gain and obesity needs some explanation. It is likely that the nature of obesity and adipose tissue accretion seen in Asians may be triggered by the consumption of high-GI diets and suggests that the drivers of obesity may not be confined to the consumption of high-fat diets alone. Our observations provide substantial public health support for the encouragement of consuming low GI meals in Asians and suggest that replacement of added sucrose in foods with isomaltulose can be a unique way in which the glycaemic response of the foods may be significantly attenuated. Thus far, the role of low GI meals has focused attention on its impact of glycaemia, though now, our study shows that it may also have an important impact on fat oxidation and possibly facilitate weight management in Asians. Our results further suggest that the use and application of isomaltulose as a part of our diet in Asia may be an even more pressing need.

## Figures and Tables

**Table 1:** Diet and meal composition and foods used to construct the low and high GI test meals provided in the study.

	Low GI Diet				High GI Diet			
	% of Total	E% CHO	E% Fat	E% Protein	% of Total	E% CHO	E% Fat	E% Protein
Dinner	35.6	68.2	11.7	20.1	35.8	70.8	11.3	17.9
	Basmati parboiled rice				Glutinous rice			
	Chicken stock				Chicken stock			
	Teriyaki chicken				Teriyaki chicken			
	Chinese spinach				Carrots			
	Fruit drink with Palatinose™				Fruit drink with sucrose			
Breakfast	16.8	77.3	9.7	13.0	16.6	83.4	7.5	9.1
	All-bran cereal Full fat milk Chamomile tea with Palatinose™				Koko Krunch cereal Low fat cheese Reduced fat milk Chamomile tea with sucrose			
Lunch	31.0	72.7	11.6	15.7	31.0	75.5	11.1	13.3
	Basmati parboiled rice				Glutinous rice			
	Chicken stock				Chicken stock			
	Chinese spinach				Carrots			
	Teriyaki chicken				Teriyaki chicken			
	Extra virgin olive oil				Extra virgin olive oil			
	Fruit drink with Palatinose™				Fruit drink with sucrose			
Snack	16.6	73.7	11.2	15.1	16.6	75.5	16.1	8.4
	Multigrain bread Strawberry jam (low GI) Chamomile tea with Palatinose™				White bread Strawberry jam Margarine Chamomile tea with sucrose			
Total	100	72.0	11.3	16.7	100	75.1	11.4	13.5

GI: glycaemic index; % of total: percentage of total daily energy intake; E% CHO: energy percentage from carbohydrate; E% Fat: energy percentage from fat; E% Protein: energy percentage from protein.

**Table 2:** Baseline subject characteristics as means ± SD and minimum to maximum range (*n* = 20).

Characteristic (*n* = 20)	Mean ± SD (Range)
Age (years)	23.8 ± 1.8 (21–29)
Height (m)	1.74 ± 0.05 (1.64–1.83)
Weight (kg)	74.2 ± 9.6 (57.1–88.5)
BMI (kg/m^2^)	24.4 ± 3.1 (18.2–28.4)
Fat percentage (%)	20.1 ± 5.4 (7.8–27.0)
Waist circumference (cm)	80.5 ± 8.2 (64.0–92.7)
Hip circumference (cm)	100.0 ± 6.3 (91.5–112.2)
Waist/hip ratio	0.80 ± 0.05 (0.67–0.89)
BMR (kcal/day)	1540 ± 143 (1307–1809)
Fasting blood glucose (mmol/L)	4.6 ± 0.5(3.6–5.5)

BMI: body mass index; BMR: basal metabolic rate.

**Table 3:** Glycaemic outcome variables for low GI and high GI treatment (mean ± SD) (*n* = 20).

	Low GI	High GI	*p*-Value
iAUC overnight after test dinner (mmol/L)	191.8 ± 168.3	318.9 ± 265.1	0.027 *
iAUC breakfast (mmol/L)	88.6 ± 53.6	177.0 ± 97.9	<0.001 ***
iAUC lunch (mmol/L)	99.0 ± 51.0	233.9 ± 116.1	<0.001 ***
iAUC snack (mmol/L)	73.9 ± 38.0	93.2 ± 62.5	0.095
10 h WBC iAUC (mmol/L)	288.0 ± 109.8	539.9 ± 290.7	<0.001 ***
24 h iAUC (mmol/L)	502.5 ± 231.4	872.6 ± 493.1	<0.001 ***
iAUC overnight after standard dinner (mmol/L)	237.0 ± 123.2	380.5 ± 297.2	0.026 *
42 h iAUC (mmol/L)	775.7 ± 333.1	1379.8 ± 804.7	0.002 **
24 h total AUC (mmol/L)	43,313 ± 3313	43,157 ± 4674	0.440
MAGE (24 h)	1.67 ± 0.53	2.68 ± 1.13	<0.001 ***

Post-prandial iAUC represents 180 min for breakfast and lunch and 120 min for snack; overnight iAUC after dinner represents 600 min of data. GI: glycaemic index; iAUC: incremental area under the curve; AUC: area under the curve; MAGE: mean amplitude of glycaemic excursion; WBC: whole body calorimetry; * *p* < 0.05, ** *p* < 0.01, *** *p* < 0.001 (paired Student’s *t*-test).

**Table 4:** Whole-body calorimeter variables for low GI and high GI treatment (mean ± SD) (*n* = 20). Incremental values are changes compared to baseline obtained before breakfast.

	Low GI	High GI	*p*-Value
Energy expenditure	1.565 ± 0.084	1.591 ± 0.097	0.19
RQ	0.907 ± 0.030	0.910 ± 0.41	0.300
Incremental RQ (10 h)	0.064 ± 0.030	0.070 ± 0.041	0.014 *
CHO oxidation (g/min)	0.268 ± 0.043	0.275 ± 0.062	0.039 *
Incremental CHO oxidation (10 h) (g/min)	0.121 ± 0.043	0.141 ± 0.063	<0.001 ***
Fat oxidation (g/min)	0.040 ± 0.018	0.038 ± 0.024	0.237
Incremental fat oxidation (10 h) (g/min)	–0.025 ± 0.018	–0.028 ± 0.025	0.052
Protein oxidation (g/min)	0.007 ± 0.003	0.007 ± 0.002	0.53

GI: glycaemic index; RQ: respiratory quotient; CHO: carbohydrate * *p* < 0.05, ** *p* < 0.01, *** *p* < 0.001 (paired Student’s *t*-test).

**Table 5:** Meal-specific changes in substrate oxidation for low GI and high GI treatments (mean ± SD) (*n* = 20). Incremental values are changes compared to meal specific baselines.

	Low GI	High GI	*p*-Value
iRQ breakfast	0.046 ± 0.034	0.054 ± 0.043	0.101
iRQ lunch	0.031 ± 0.024	0.074 ± 0.041	<0.001 ***
iRQ snack	0.006 ± 0.013	–0.011 ± 0.017	<0.001***
iCHO oxidation breakfast (g/min)	0.086 ± 0.039	0.102 ± 0.058	0.013 *
iCHO oxidation lunch (g/min)	0.059 ± 0.029	0.126 ± 0.056	<0.001 ***
iCHO oxidation snack (g/min)	0.005 ± 0.018	−0.013 ± 0.027	0.003 **
iFat oxidation breakfast (g/min)	−0.015 ± 0.021	−0.021 ± 0.025	0.026 *
iFat oxidation lunch (g/min)	−0.014 ± 0.015	−0.041 ± 0.027	<0.001 ***
iFat oxidation snack (g/min)	−0.003 ± 0.009	0.002 ± 0.011	0.013 *
iRQ breakfast	0.046 ± 0.034	0.054 ± 0.043	0.101

GI: glycaemic index; i: incremental; RQ: respiratory quotient; CHO: carbohydrate; * *p* < 0.05, ** *p* < 0.01, *** *p* < 0.001 (paired Student’s *t*-test).
